# The impact of poverty transitions on frailty among older adults in South Korea: findings from the Korean longitudinal study of ageing

**DOI:** 10.1186/s12877-020-01522-x

**Published:** 2020-04-15

**Authors:** Hin Moi Youn, Hyeon Ji Lee, Doo Woong Lee, Eun-Cheol Park

**Affiliations:** 1 Department of Public Health, Yonsei University Graduate School, 50 Yonsei-ro, Seodaemun-gu, Seoul, 03722 Republic of Korea; 2 Institute of Health Services Research, Yonsei University, 50 Yonsei-ro, Seodaemun-gu, Seoul, 03722 Republic of Korea; 3 Department of Preventive Medicine and Institute of Health Services Research, Yonsei University College of Medicine, 50 Yonsei-ro, Seodaemun-gu, Seoul, 03722 Republic of Korea

**Keywords:** Frailty, Frail, Poverty, Elderly, Socioeconomic status, Healthy aging

## Abstract

**Background:**

Frailty is an emerging public health concern among aging populations. Although socioeconomic status is a well-known contributor to frailty, there is limited research investigating the effects of poverty on frailty. This study aimed to examine the association between poverty transitions and frailty prevalence in older adults.

**Methods:**

Data were collected from the six-wave Korean Longitudinal Study of Ageing (2006–2016). A total of 9263 middle-aged and older adults were included in the analysis. Poverty was defined as being below 50% of the median household income based on the equivalized household. Frailty was measured using an instrument comprising items on physical phenotype (grip strength) and psychological (exhaustion) and social aspects (isolation). Analyses using generalized estimating equations were conducted to estimate the relationship between poverty transition and frailty status.

**Results:**

Among the 9263 respondents, 9.4% of the male respondents (*n* = 388) and 13.6% of the female respondents (*n* = 700) were frail. After controlling for covariates, female participants who transitioned into poverty (OR = 1.31, 95% CI: 1.02–1.69) and persistently remained in poverty (OR = 1.36, 95% CI: 1.10–1.68) showed increased odds of frailty in the follow-up year. We did not find significant results in the male participants.

**Conclusions:**

The findings suggest that those who experience poverty transitions, enter poverty, and remain in poverty persistently are at higher risk of frailty. To improve age-related health status among the elderly, interventions aiming to prevent and reduce frailty among the elderly should target individuals who are more vulnerable to the negative effects of frailty.

## Background

Population aging is a global phenomenon. Increased life expectancy and low birth rates have contributed to the world’s aging population, and virtually every country is experiencing growth in the proportion of older people in their population [1]. South Korea has one of the fastest aging populations—the proportion of people aged 65 or older is projected to reach 46.5% by 2067, rendering it the world’s most aged country [2]. Although increased life expectancy is a major achievement, it presents challenges regarding the burden of age-related diseases, such as health costs and social care. The prevention and treatment of frailty has recently received increasing attention with respect to the promotion of healthier aging among the elderly [3]. Frailty develops as a consequence of age-related decline in multiple physiological systems, which collectively results in vulnerability to adverse health status changes triggered by stressor events [4]. Frail individuals are at a greater risk of adverse health outcomes such as falls, incident disability, institutionalization, hospitalization, and death, and have a greater need for healthcare [5]. Thus, frailty can have consequences for quality of life, health, and welfare systems.

The incidence of frailty may vary diversely among individuals in that some are more vulnerable to stressors whereas others are resilient. Frailty in the elderly is a multidimensional syndrome that involves the interaction of biological, psychological, and social factors [4]. To date, various studies have shown that socioeconomic status (including education, occupation, and income) is an important contributor to the disparities in frailty among the elderly [5–9]. Although it is well recognized that poverty has important implications for health, few studies have focused on poverty and its effect on frailty [10]. Moreover, there has been limited research on the effect of changes in poverty status over time. Because frailty development is influenced by the aging-related accumulation of deficits, longitudinal assessment can provide a more robust understanding of the extent to which poverty is associated with frailty.

While a higher prevalence of elderly poverty is a global phenomenon, South Korea has the highest poverty rate of people aged over 65 among the Organization for Economic Co-operation and Development (OECD) countries. In 2017, the country’s elderly poverty rate, which indicates the proportion of senior citizens earning below 50% of the overall median income, reached 43.8%, whereas the average poverty rate in the OECD was 13.5% [11]. Therefore, better understanding of the effects of poverty on frailty is necessary for the development of intervention strategies aimed at preventing and reducing frailty and its burden on individuals, especially those in poverty. In this study, we sought to assess the prevalence of frailty in middle-aged and older adults and examine the impact of poverty transition on frailty.

## Methods

### Data source and study population

Data were collected from the Korean Longitudinal Study of Ageing (KLoSA), which was conducted in 2006, 2008, 2010, 2012, 2014, and 2016. The KLoSA is a large-scale, longitudinal survey of the population aged 45 and older living in households selected by multistage stratified probability sampling to ensure national representativeness. It was designed to help develop policies to address health and social issues that emerged because of rapid population aging. In the 2006 baseline survey, the original sample of 10,254 respondents completed interviews by well-trained interviewers. The household response rate was 70.7% and the individual response rate within households was 75.4%. This survey was followed up with 8875, 8229, and 7813 respondents in 2008, 2010, and 2012, respectively. A refreshment sample of 920 individuals born in 1962 or 1963 was introduced in 2014 and was included in the 2014 and 2016 waves. The combined sample included 8387 respondents in 2014 and 7893 in 2016 [12]. After excluding those with missing data and those who were unable to follow up, 9263 respondents were included in the present sample.

### Measures

#### Poverty transitions

The variable of interest was the transition of poverty status across time. We employed a relative measure of poverty, defining it as earning below 50% of the median household income based on the equivalized household. The value of the poverty line was set for each year (2006, 2008, 2010, 2012, 2014, 2016) based on data from Statistics Korea. The KLoSA contains detailed information about the different types of income that comprise aggregate income, including earned income, asset income, public transfer income, financial support, and other types of income. Total household income is the sum of the incomes of all household members living together, including the respondent. The household income reported by the representative member was assigned to all the other members such that that the total amount of household income had the same value across all household members [12]. In the present study, we used equivalized household income, which considers the square root of the number of household members. The current equivalized household income of all respondents in the sample was allocated into poverty and non-poverty groups based on the previously defined poverty line. Poverty transition was measured as change in poverty status in a previous year (Y-1) and the subsequent year (Y0). We categorized the respondents into four groups: non-poverty to non-poverty (NN, persistence of non-poverty), poverty to non-poverty (PN, exiting poverty), non-poverty to poverty (NP, transition to poverty), and poverty to poverty (PP, persistence of poverty) [13].

#### Frailty

We used a broader definition of frailty that includes physical phenotype and social and psychological aspects. The frailty instrument consists of items measuring weakness of grip strength, exhaustion, and social isolation. It was developed to assess the risks of adverse health outcomes such as disability, institutionalization, and mortality of older adults with high predictive validity, discrimination, and calibration ability. The validity of the frailty instrument has been reported elsewhere [14]. Weakness was measured using grip strength (< 24 kg for men and < 15 kg for women). Exhaustion was evaluated by self-reporting either the feeling that every task required effort or that they could not “get going” in the preceding week. Isolation was assessed by asking about participation in meetings or group activities. The scale scores ranged from 0 to 3 and were categorized as frail (≥ 2), pre-frail (≥ 1), and robust (0) [15]. In this study, we grouped participants into two categories: frail (≥ 2) and non-frail (≤ 1).

#### Covariates

Demographic, socioeconomic, and health-related factors were included in the study. The demographic variables were sex, age (45–64, 65–74, 75 or older), marital status (with spouse, without spouse), and number of household members (1, 2, 3, or more). The socioeconomic variables included educational level (elementary school or below, middle/high school, college, or above), household income (quantiles), current economic activity (active, inactive), region (metropolitan, urban, rural), and health insurance (national health insurance, medical aid). The health-related factors included smoking (yes, no), drinking (yes, no), perceived health status (healthy, average, unhealthy), and presence of chronic diseases (yes, no). Chronic diseases included hypertension, diabetes, malignant tumor, liver disease, cardiovascular disease, cerebrovascular disease, psychiatric disorders, and rheumatoid arthritis. We used indicators of individuals’ functional and cognitive status, including activities of daily living [independent, needs help/difficulty with activities of daily living (ADL)], instrumental activities of daily living [independent, needs help/difficulty with instrumental ADL (IADL)], and cognitive impairment (yes, no). Cognitive impairment was measured using the Korean Mini-Mental State Examination (K-MMSE), which includes 11 items in seven categories of cognitive functions (orientation of time and place, registration, attention and calculation, recall, language, and visual construction). The total score range from 0 to 30, and higher scores indicate better cognitive function. The validity of the K-MMSE has been reported elsewhere [16]. We followed the conventional classification criteria and categorized scores as indicating normal cognitive function (K-MMSE ≥24) and mild to severe cognitive impairment (K-MMSE ≤23). Frailty status in the previous year was included to take account of its contribution to frailty in the follow-up year.

### Statistical analysis

The distribution of general characteristics was calculated at baseline. Differences in baseline characteristics between non-frail and frail respondents were determined using *χ*^2^ tests. To evaluate repeatedly measured individuals, PROC GENMOD was used to employ a generalized estimating equation (GEE) for repeated measure analysis. We evaluated whether the probability of frailty changed after poverty transitions over two consecutive years (between 2006 and 2008, 2008–2010, 2010–2012, 2012–2014, or 2014–2016). Furthermore, subgroup analyses stratified by age, marital status, current economic activity, region, presence of chronic diseases, and cognitive impairment were performed to examine the association between poverty transitions and frailty after adjusting for covariates. All analyses were conducted using SAS software, version 9.3 (SAS Institute, Cary, NC).

## Results

**Fig. 1:**
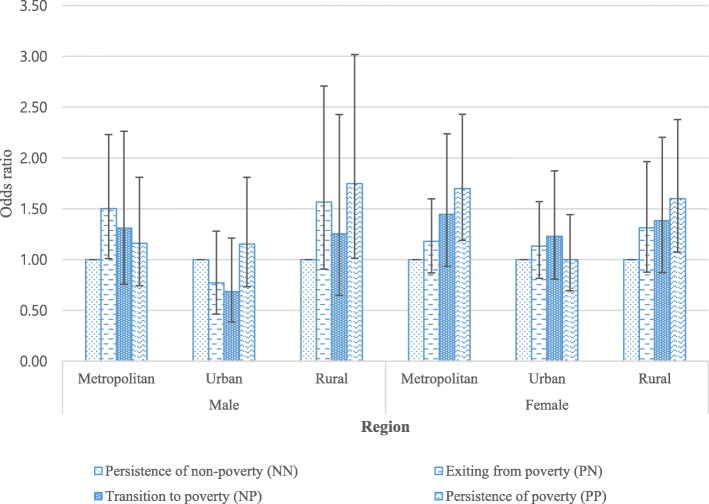
Results of subgroup analysis of poverty transitions to frailty stratified by region. Control variables include age, sex, marital status, number of household, educational level, current economic activity, health insurance type, household income, smoking, drinking, perceived health status, chronic disease, disability, ADL, IADL, cognitive impairment, frailty in the previous year. 95% confidence interval

Table 1 presents the baseline characteristics of the study population. Among the total 9263 participants, 44.4% (4115) were men and 55.6% (5148) were women. Overall, 388 (9.4%) men and 700 (13.6%) women were frail. With regard to poverty status, 2111 (51.3%) men and 2894 (56.2%) women were below the poverty threshold. Among those in poverty, women showed a greater proportion of frailty (18.8%) than men (15.1%). Across age groups, the oldest group had the highest proportion of frailty in both men (29.8%) and women (36.6%). Furthermore, the lower income quantiles showed smaller proportions of frail individuals among both men and women (15.6 and 20.3% for men and women in Quantile 1, respectively). Table 2 shows the results of the GEE model for the impact of poverty transitions on frailty. Among women, those who were not in poverty in the previous year but entered poverty in the subsequent year (PN) (OR = 1.31, 95% CI: 1.02–1.69) and those who were persistently in poverty (PP) (OR = 1.36, 95% CI: 1.10–1.68) showed increased odds of frailty compared with those who were persistently not in poverty (NN). Among men, there was no statistically significant relationship between poverty transitions and frailty. However, although not statistically significant, those who were persistently in poverty showed the highest odds of frailty (OR = 1.23, 95% CI: 0.94–1.62). Men aged 75 years or older had the highest odds of frailty (OR = 2.40, 95% CI: 1.87–3.09) while women aged between 65 and 74 had the highest odds of frailty (OR = 1.24, 95% CI: 1.06–1.46). Significant relationships were found between household income and frailty. Those in the highest quantile showed the lowest odds of frailty among both men (OR = 0.41, 95% CI: 0.27–0.62) and women (OR = 0.71, 95% CI: 0.52–0.97). Frailty in the previous year was significantly associated with frailty in the subsequent year for both men (OR = 3.61, 95% CI: 2.96–4.40) and women (OR = 3.41, 95% CI: 2.98–3.89). Figure 1 presents the results for the subgroup analysis of the association between poverty transitions and frailty stratified by region. The results show that, compared to individuals living in urban area, those living in rural and metropolitan areas have greater odds of being frailty. In addition, women show a graded association between poverty transitions and frailty, where persistently remained in poverty had the highest odds in metropolitan (OR = 1.70, 95% CI: 1.19–2.43) and rural (OR = 1.60, 95% CI: 1.07–2.38).

**Table 1:** General characteristics of the study population (2006 baseline)

Variables	Frailty^**a**^													
	Male						***p*** value	Female						***p*** value
	Total		Yes		No			Total		Yes		No		
	N	%	N	%	N	%		N	%	N	%	N	%	
	4115	100.0	388	9.4	3727	90.6		5148	100.0	700	13.6	4448	86.4	
**Current relative poverty**^**b**^							<.0001							<.0001
Non-poverty	2004	48.7	69	3.4	1935	96.6		2254	43.8	155	6.9	2099	93.1	
Poverty	2111	51.3	319	15.1	1792	84.9		2894	56.2	545	18.8	2349	81.2	
**Age**							<.0001							<.0001
45–64	2554	62.1	104	4.1	2450	95.9		3177	61.7	167	5.3	3010	94.7	
65–74	1125	27.3	154	13.7	971	86.3		1285	25.0	282	21.9	1003	78.1	
≥ 75	436	10.6	130	29.8	306	70.2		686	13.3	251	36.6	435	63.4	
**Marital status**							<.0001							<.0001
With spouse	3788	92.1	307	8.1	3481	91.9		3571	69.4	299	8.4	3272	91.6	
Without spouse	327	7.9	81	24.8	246	75.2		1577	30.6	401	25.4	1176	74.6	
**No. of household members**							<.0001							<.0001
1	139	3.4	36	25.9	103	74.1		632	12.3	156	24.7	476	75.3	
2	1706	41.5	203	11.9	1503	88.1		1820	35.4	240	13.2	1580	86.8	
≥ 3	2270	55.2	149	6.6	2121	93.4		2696	52.4	304	11.3	2392	88.7	
**Educational level**							<.0001							<.0001
Elementary school graduate or lower	1260	30.6	252	20.0	1008	80.0		2886	56.1	619	21.4	2267	78.6	
Middle/high school graduate	2125	51.6	110	5.2	2015	94.8		1998	38.8	77	3.9	1921	96.1	
College graduate or higher	730	17.7	26	3.6	704	96.4		264	5.1	4	1.5	260	98.5	
**Current economic activity**							<.0001							<.0001
Active	2441	59.3	84	3.4	2357	96.6		1298	25.2	90	6.9	1208	93.1	
Inactive	1674	40.7	304	18.2	1370	81.8		3850	74.8	610	15.8	3240	84.2	
**Region**							<.0001							0.0014
Metropolitan	1800	43.7	152	8.4	1648	91.6		2353	45.7	310	13.2	2043	86.8	
Urban	1390	33.8	113	8.1	1277	91.9		1673	32.5	202	12.1	1471	87.9	
Rural	925	22.5	123	13.3	802	86.7		1122	21.8	188	16.8	934	83.2	
**Health insurance type**							<.0001							<.0001
NHI^c^	3907	94.9	323	8.3	3584	91.7		4838	94.0	592	12.2	4246	87.8	
Medical aid	208	5.1	65	31.3	143	68.8		310	6.0	108	34.8	202	65.2	
**Household income**^**d**^							<.0001							<.0001
Quantile 1 (low)	1695	41.2	265	15.6	1430	84.4		2393	46.5	485	20.3	1908	79.7	
Quantile 2	902	21.9	80	8.9	822	91.1		1078	20.9	121	11.2	957	88.8	
Quantile 3	551	13.4	19	3.4	532	96.6		599	11.6	39	6.5	560	93.5	
Quantile 4	439	10.7	13	3.0	426	97.0		504	9.8	31	6.2	473	93.8	
Quantile 5 (high)	528	12.8	11	2.1	517	97.9		574	11.1	24	4.2	550	95.8	
**Smoking**							0.6453							<.0001
No	1593	38.7	146	9.2	1447	90.8		4956	96.3	642	13.0	4314	87.0	
Yes	2522	61.3	242	9.6	2280	90.4		192	3.7	58	30.2	134	69.8	
**Drinking**							0.2594							0.0445
No	986	24.0	102	10.3	884	89.7		4035	78.4	569	14.1	3466	85.9	
Yes	3129	76.0	286	9.1	,843	90.9		1113	21.6	131	11.8	982	88.2	
**Perceived health status**							<.0001							<.0001
Healthy	680	16.5	14	2.1	666	97.9		565	11.0	12	2.1	553	97.9	
Average	1685	40.9	48	2.8	1637	97.2		1669	32.4	86	5.2	1583	94.8	
Unhealthy	1750	42.5	326	18.6	1424	81.4		2914	56.6	602	20.7	2312	79.3	
**Chronic disease**^**e**^							<.0001							<.0001
No	2349	57.1	139	5.9	2210	94.1		2598	50.5	213	8.2	2385	91.8	
Yes	1766	42.9	249	14.1	1517	85.9		2550	49.5	487	19.1	2063	80.9	
**Disability**							<.0001							<.0001
No	3801	92.4	314	8.3	3487	91.7		4967	96.5	653	13.1	4,14	86.9	
Yes	314	7.6	74	23.6	240	76.4		181	3.5	47	26.0	134	74.0	
**ADL**^**f**^							<.0001							<.0001
Independent	4022	97.7	339	8.4	3683	91.6		5011	97.3	635	12.7	4376	87.3	
Needs help/difficulty with ADL	93	2.3	49	52.7	44	47.3		137	2.7	65	47.4	72	52.6	
**IADL**^**g**^							<.0001							<.0001
Independent	3546	86.2	269	7.6	3277	92.4		4673	90.8	524	11.2	4149	88.8	
Needs help/difficulty with IADL	569	13.8	119	20.9	450	79.1		475	9.2	176	37.1	299	62.9	
**Cognitive impairment**^**h**^							<.0001							<.0001
No	3572	86.8	226	6.3	3346	93.7		3715	72.2	277	7.5	3438	92.5	
Yes	543	13.2	162	29.8	381	70.2		1433	27.8	423	29.5	1010	70.5	

^a^ The frailty instrument consists of grip strength, exhaustion, and social isolation (frail ≥2, non-frail ≤1)

^b^ Relative poverty line: 50% of median household income based on the equivalized household income

^c^ NHI: National Health Insurance (employee and self-employee insured)

^d^ Participants’ current equivalized household income level was allocated into quantile groups based on the data from Statistics Korea

^e^ Chronic diseases include hypertension, diabetes, malignant tumor, liver disease, cardiovascular disease, cerebrovascular disease, psychiatric disorders, and rheumatoid arthritis disease

^f^ ADL: Activities of daily living

^g^ IADL: Instrumental activities of daily living

^h^ K-MMSE (Korean Mini-Mental State Examination): normal cognitive function (K-MMSE≥24) and mild to severe cognitive impairment (K-MMSE≤23)

**Table 2:** Association between poverty transitions and frailty: the results of GEE analysis

Variables	Frailty^**a**^							
	Male				Female			
	OR^i^	95% CI^i^			OR^i^	95% CI^i^		
**Poverty transition**^b^								
Persistence of non-poverty (NN)	1.00				1.00			
Exiting poverty (PN)	1.22	(0.93	–	1.59)	1.16	(0.96	–	1.41)
Transition to poverty (NP)	1.03	(0.74	–	1.44)	1.31	(1.02	–	1.69)
Persistence of poverty (PP)	1.23	(0.94	–	1.62)	1.36	(1.10	–	1.68)
**Age**								
45–64	1.00				1.00			
65–74	1.40	(1.12	–	1.76)	1.24	(1.06	–	1.46)
≥ 75	2.40	(1.87	–	3.09)	1.81	(1.50	–	2.17)
**Marital status**								
With spouse	1.00				1.00			
Without spouse	1.62	(1.28	–	2.05)	1.29	(1.12	–	1.49)
**No. of household members**								
1	1.00				1.00			
2	0.93	(0.71	–	1.23)	1.12	(0.95	–	1.31)
≥ 3	0.98	(0.74	–	1.31)	1.13	(0.96	–	1.32)
**Educational level**								
Elementary school graduate or lower	1.00				1.00			
Middle/high school graduate	0.83	(0.70	–	0.99)	0.70	(0.60	–	0.82)
College graduate or higher	0.65	(0.48	–	0.88)	0.58	(0.33	–	1.00)
**Current economic activity**								
Active	1.00				1.00			
Inactive	1.40	(1.17	–	1.67)	1.11	(0.97	–	1.28)
**Region**								
Metropolitan	1.00				1.00			
Urban	1.18	(0.98	–	1.42)	1.20	(1.05	–	1.38)
Rural	1.00	(0.82	–	1.21)	1.03	(0.90	–	1.18)
**Health insurance type**								
NHI^c^	1.00				1.00			
Medical aid	1.35	(1.02	–	1.78)	1.49	(1.22	–	1.81)
**Household income**^d^								
Quantile 1 (low)	1.00				1.00			
Quantile 2	0.80	(0.66	–	0.96)	0.76	(0.66	–	0.88)
Quantile 3	0.58	(0.41	–	0.82)	0.85	(0.65	–	1.10)
Quantile 4	0.63	(0.43	–	0.92)	0.92	(0.69	–	1.23)
Quantile 5 (high)	0.41	(0.27	–	0.62)	0.71	(0.52	–	0.97)
**Smoking**								
No	1.00				1.00			
Yes	1.24	(1.05	–	1.46)	1.54	(1.21	–	1.97)
**Drinking**								
No	1.00				1.00			
Yes	0.96	(0.80	–	1.16)	1.03	(0.90	–	1.18)
**Perceived health status**								
Healthy	1.00				1.00			
Average	0.99	(0.68	–	1.45)	0.91	(0.62	–	1.35)
Unhealthy	2.16	(1.47	–	3.16)	2.78	(1.91	–	4.06)
**Chronic disease**^**e**^								
No	1.00				1.00			
Yes	1.12	(0.95	–	1.32)	1.11	(0.97	–	1.27)
**Disability**								
No	1.00				1.00			
Yes	0.94	(0.46	–	1.94)	0.84	(0.48	–	1.47)
**ADL**^**f**^								
Independent	1.00				1.00			
Needs help/difficulty with ADL	1.92	(1.18	–	3.12)	1.79	(1.29	–	2.49)
**IADL**^**g**^								
Independent	1.00				1.00			
Needs help/difficulty with IADL	1.30	(1.06	–	1.59)	1.97	(1.64	–	2.37)
**Cognitive impairment**								
No	1.00				1.00			
Yes	2.82	(2.40	–	3.31)	2.32	(2.06	–	2.62)
**Frailty in the previous year**								
No	1.00				1.00			
Yes	3.61	(2.96	–	4.40)	3.41	(2.98	–	3.89)

^a^ The frailty instrument consists of grip strength, exhaustion, and social isolation (frail ≥2, non-frail ≤1)

^b^ Relative poverty line: 50% of median household income based on the equivalized household income

^c^*NHI* National Health Insurance (employee and self-employee insured)

^d^ Participants’ current equivalized household income level was allocated into quantile groups based on the data from Statistics Korea

^e^ Chronic diseases include hypertension, diabetes, malignant tumor, liver disease, cardiovascular disease, cerebrovascular disease, psychiatric disorders, and rheumatoid arthritis disease

^f^*ADL* Activities of daily living

^g^*IADL* Instrumental activities of daily living

^h^*K-MMSE (Korean Mini-Mental State Examination)* normal cognitive function (K-MMSE ≥24) and mild to severe cognitive impairment (K-MMSE ≤23)

^i^OR: odds ratio; 95% CI: confidence interval

## Discussion

In this study, we examined whether transitions in poverty status are associated with frailty in middle-aged and older adults in South Korea. As aforementioned, about a half of the Korean elderly population is living in poverty. In our study, we found that more than half of the individuals aged over 45 years were below the poverty threshold. Poverty is a well-known socioeconomic determinant that is intertwined with health. We attempted to shed light on how poverty corresponds to frailty, that is, the adverse health outcomes of accumulated risk factors over the course of a lifetime. The prevalence of frailty in Korea has been reported to range from 2.5 to 31.7% depending on the study population and components of each frailty scale [15, 17, 18]. In our study, we used a frailty instrument that measures physical, psychological, and social domains, which can contribute to frailty independently or interactively [19]. The prevalence of frailty was 11.7% (men 9.4% and women 13.6%). The results indicated that experiencing poverty significantly increased the probability of frailty compared to persistently remaining in non-poverty. The findings are in line with those of previous studies that found that poverty is associated with frailty or poorer physical, psychological, and cognitive functioning [10, 20, 21] However, significant relationships between poverty transitions and frailty were observed only in women. In general, women are more likely to be at risk related to overall health because of several factors, including age-related diseases, low socioeconomic status, and low activity level. Our results are in accordance with those of previous studies that found women are frailer than men [5, 17, 22, 23]. The finding that poverty affects women and men unequally offers a direction for targeted interventions to prevent or manage symptoms of frailty. It has been shown that the probability of frailty increases with persistent poverty over time [20, 24]. Poverty leads to various health risks such as less knowledge about healthy behaviors, lower access to health services, and environmental risks for illness and disability [25]. Given that frailty develops because of accumulated deficits over time, sustained exposure to risks due to poverty will increase the prevalence of frailty. Furthermore, those who transitioned into poverty in the follow-up year showed an increased probability of frailty as well. A study found that income change, particularly income loss, is significantly associated with health [24]. There could be several reasons for poverty transition; however, sudden or unpredictable financial loss would be particularly damaging to health. For example, if poverty transitions occur because of unexpected job loss, it could cause not only financial loss but also acute disappointment, which could result in depression or social isolation [26]. It is not surprising that age is strongly associated with frailty. The present findings showed a graded association with increasing age, which is supported by previous studies [6, 27, 28]. The findings from the subgroup analysis demonstrated that middle-aged women did not show a significant association with frailty, suggesting that older women are more vulnerable to poverty transitions. As has been well studied in the literature [29, 30], socioeconomic variables including education and income levels, which are related to poverty, are also associated with frailty. For both men and women, being unmarried or living without spouse was associated with frailty. With older age, those who experienced bereavement or widowhood reported feeling more depressive symptoms and lower social ties, which may negatively affect their health [31]. Our findings were consistent with those of a previous study which found that being cognitively impaired increased the probability of frailty [32]. Furthermore, those who reported being dependent for their ADL and IADL had a higher probability of frailty [18, 33, 34].

Although these findings elucidate how transitions in poverty status affect frailty, they should be interpreted with caution because of several limitations. First, the frailty scale used in the KLoSA was developed and validated only in Korea. However, it measures physical, psychological, and social determinants, thereby offering a broader approach to explaining frailty. Second, although our study was based on longitudinal data with repeated observations at the individual level over a period, we could not determine a perfect causal relationship between poverty transitions and frailty. Third, measurement errors due to recall bias might exist because of subjective and inaccurate responses by the respondents. Despite these limitations, our study has several strengths. The KLoSA is a South Korean panel study focusing on the elderly that has been verified by experts to have statistical validity and national representativeness. We measured poverty prevalence based on actual data calculated by Statistics Korea for greater reliability.

Based on the present findings, those who transition into poverty and stay persistently in poverty are at high risk of frailty, particularly women aged over 65 years. Previous studies have shown that better management and intervention may prevent the progress of frailty or increase the chances of recovering from frailty [35]. Providing care to frail individuals is difficult because of their vulnerability to deterioration, complex comorbidities, and increased social needs [36, 37]. Furthermore, frailty is influenced by multiple factors and their complex interactions, which accumulate over time. Thus, future research on the various aspects of frailty and how they are influenced by socioeconomic and cultural determinants would provide a better understanding of frailty in older age.

## Conclusions

To our knowledge, this is one of the few studies investigating the effects of poverty transitions on frailty. Our study aims to expand the knowledge regarding frailty in socioeconomically vulnerable groups. The findings suggest that experiencing poverty transitions, entering poverty, and persistently being in poverty increase the risk of frailty. This study can contribute to the development of intervention strategies to better identify frail individuals who may be at greater risk of negative effects on health.

## Data Availability

The data used in the study are available at https://survey.keis.or.kr/eng/klosa/databoard/List.jsp

## Abbreviations

ADL: Activities of daily living
GEE: Generalized estimating equation
IADL: Instrumental activities of daily living
OECD: Organization for Economic Co-operation and Development
KLoSA: Korean Longitudinal Study of Ageing
